# Metabolic Engineering for Valorization of Agri- and Aqua-Culture Sidestreams for Production of Nitrogenous Compounds by *Corynebacterium glutamicum*

**DOI:** 10.3389/fmicb.2022.835131

**Published:** 2022-02-08

**Authors:** Volker F. Wendisch, K. Madhavan Nampoothiri, Jin-Ho Lee

**Affiliations:** ^1^Genetics of Prokaryotes, Faculty of Biology and Center for Biotechnology, Bielefeld University, Bielefeld, Germany; ^2^Microbial Processes and Technology Division, Council of Scientific and Industrial Research–National Institute for Interdisciplinary Science and Technology, Thiruvananthapuram, India; ^3^Department of Food Science & Biotechnology, Kyungsung University, Busan, South Korea

**Keywords:** nitrogen valorization, agriculture sidestreams, aquaculture sidestreams, *Corynebacterium*, biorefinery, circular bioeconomy, metabolic engineering

## Abstract

*Corynebacterium glutamicum* is used for the million-ton-scale production of amino acids. Valorization of sidestreams from agri- and aqua-culture has focused on the production of biofuels and carboxylic acids. Nitrogen present in various amounts in sidestreams may be valuable for the production of amines, amino acids and other nitrogenous compounds. Metabolic engineering of *C. glutamicum* for valorization of agri- and aqua-culture sidestreams addresses to bridge this gap. The product portfolio accessible *via C. glutamicum* fermentation primarily features amino acids and diamines for large-volume markets in addition to various specialty amines. On the one hand, this review covers metabolic engineering of *C. glutamicum* to efficiently utilize components of various sidestreams. On the other hand, examples of the design and implementation of synthetic pathways not present in native metabolism to produce sought after nitrogenous compounds will be provided. Perspectives and challenges of this concept will be discussed.

## Graphical Abstract

Strategy to valorize non-utilized nitrogen from sidestreams by producing nitrogen-containing products.

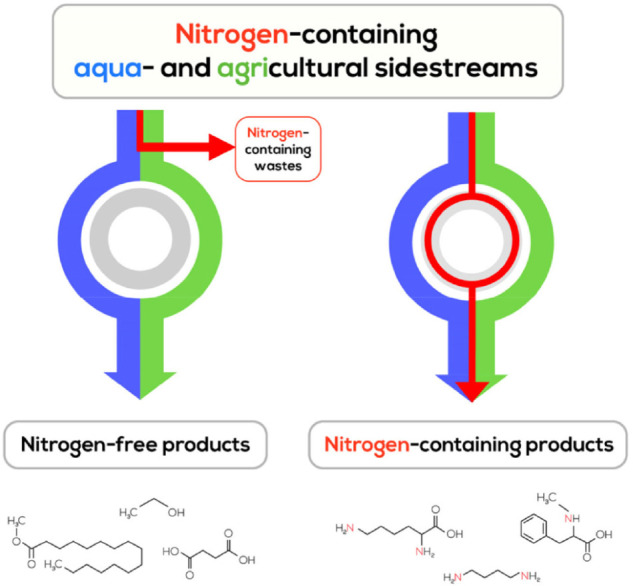

## Introduction

In this review, we focus on metabolic engineering of *Corynebacterium glutamicum* for production of nitrogenous compounds from agri- and aqua-culture sidestreams. To achieve a circular bioeconomy, we need to manage renewable bioresources, reduce dependence on fossil-based and other non-sustainable resources, and avoid food- and feed-competitive raw materials. In that sense, sidestreams from agri- and aqua-culture are valuable resources and a number of approaches for their use have been reviewed previously, e.g., regarding lignin valorization (Wendisch et al., 2018), lignocellulosic feedstocks (Chen et al., 2019) or food wastes (Lee and Wendisch, 2017b). Metabolic engineering and enzyme screening and engineering were instrumental to achieve efficient bioprocesses based on sidestreams or their constituents (Lee and Wendisch, 2017b). The microbes utilizing these sidestreams produce a variety of products. Hitherto, the product scope was narrow, focusing on specific compounds such as organic acids to chemically produce polyesters or alcohols for use as biofuels (Vivek et al., 2021). Strikingly, most of the target compounds lack nitrogen although the aqua- and agricultural sidestreams typically contain a nitrogenous fraction. Thus, the production of nitrogenous value-added compounds from these sidestreams is highly relevant to make use of the full potential of biorefineries in the circular bioeconomy concept.

In this regard, the biotechnological workhorse *C. glutamicum* is relevant as it is used for the million-ton-scale production of amino acids (Wendisch, 2020) and it has been engineered for production of other nitrogenous compounds such as diamines for large-volume markets in addition to various specialty amines (Mindt et al., 2020). Moreover, a flexible feedstock concept has been realized for this bacterium enabling it to efficiently use various substrates present in aqua- and agri-sidestreams or their respective hydrolyzates. Recent developments, perspectives and challenges of production of nitrogenous compounds from agri- and aqua-culture sidestreams by metabolically engineered *C. glutamicum* will be discussed.

## Protein-Rich Byproducts From Agri- and Aqua-Culture Sidestreams

Biorefineries typically target to production of nitrogen-free value-added products from agri- and aqua-culture sidestreams, but do not make full use of the nitrogen fraction. Wastes from food in general has a distinctive configuration of nearly 30–60 wt% starch, 10–40 wt% lipids, and 5–10 wt% protein (Pleissner and Lin, 2013). Huge amount of protein, from plant and animal derivation, could be a sensuous attractive raw material for the value addition and it has to be better explored for various bioprocessing. Plant and animal parts which were uneatable were removed during the process of harvesting and post harvesting and other nutrient-rich wastes, such as manure and dead-stock all contain high levels of recoverable protein and currently, only a portion of it finds repeated use as animal feeds or animal feed constituent.

Even though the consumable portion of these protein-rich by-products could be used for recuperation of essential amino acids for human consumption, or as is for use in animal feeds, higher value solicitation for inedible and non-essential amino acids can be utilized as a feedstock for protein-based materials and other platform chemicals. Apart from the more specific uses of protein hydrolyzates, the agro-industrial by-products also find value addition through use in biological processes. Many of such products could be ideal media for several microorganisms competent of producing a diversification of bioactive metabolites, antimicrobial compounds and enzymes.

### Availability of Various Nitrogen-Containing Sidestreams

Among the plant-derived additives, grains, such as maize, barley, sorghum, oats, and wheat, and these grain by-products include corn gluten meal, brewers and distiller’s grains, malt sprouts, brewer’s yeast, and wheat mill feed represent a considerable portion (Naik et al., 2010). The inedible and non-essential amino acids obtained from these by-products can supply a feedstock for protein-based plastics, biopesticides or commodity organic compounds (Scott et al., 2007). Similarly, oil production by-products such as oil meals and press cakes from processing oilseeds, like soybean, canola, sunflower seed, linseed, palm kernel and others, are also notable feed ingredients. Some of these oil seeds have high crude protein content such as soybean meal (44.4–53.8%), cotton scotton seed meal (34–44%), castor seed cake (31–36%), canola seed meal (35–37%), pea nut meal (50–51%), etc. Further, various nuts, seeds, and their by-products, such as hulls and seed screenings, legume by-products, such as bean straw meal and hulls also contain high levels of protein (∼20–30%). Hicks and Verbeek (2017) broadly reviewed the typical protein content and global production of some of the protein meals produced from the agricultural industry. Dried ruminant waste (manure), dried poultry waste, dried poultry litter, dried swine waste, undried processed animal waste products, and processed animal waste derivatives are some of the animal waste, which has been used as a feed ingredient. Similarly, fishmeal, dried fish soluble, crab meal, shrimp meal, fish protein concentrate, and other fish by-products are some of the feed ingredients derived from marine origin. The protein content in dried manure ranges from 12 to 18 wt% for cattle, 28 to 48 wt% for poultry, and 22 to 25 wt% for pigs (Chen et al., 2003), making it a supreme source of valuable protein and nutrients.

Slaughterhouse wastes contain, e.g., blood, fat, bones, inedible parts. Protein recovery from inedible tissues is an essential step in valorization of slaughterhouse waste and the general practice is solubilizing the protein in an aqueous medium with the help of heat, chemicals, and enzymes individually or in amalgam followed by work up of the hydrolyzate to recover partially hydrolyzed protein (Adhikari et al., 2018).

As a potential renewable and nitrogen-rich feedstock, tons of shrimp shell waste (counting head, exoskeleton and walking appendages) are generated from the increasing consumption of seafood and most of them is either dumped or land filled. There is massive opportunity to introduce emerging methods and technologies in seafood waste application to produce value-added chemicals and materials. Similarly, seafood waste utilization will create benefits in both environmental and economic aspects. A crustacean shell in general contains 20–40% protein. It is reported that wastes from all the chelipede genotypes of *M. vollenhovenii* showed advisable protein content between 40.53 ± 0.56% DM and 53.73 ± 0.24% DM (Oyebola and Yildiz, 2016). High levels of protein, encompassing 40–50% dry matter, were found in the shell wastes of shrimp, 44–52% crude protein appeared in shrimp’s discard, and 50.3% crude protein was reported for processing waste powder of *M. rosenbergii* (Shahidi, 1994). This high level of protein indicates the potential for a wider use of the wastes from the *M. vollenhovenii* genotypes as proteinaceous food source for humans. Using a reformed chitinase (‘Chit46-CBM3’), (Deng et al., 2020) come out with a one-step shrimp shell processing method. The method could transform 46.5% of chitin in shrimp shells to chitin oligomers by hydrolysis in 12 h which resulted in partial protein release accompanied chitin hydrolysis. The hydrolyzate includes 8.8 g/L chitin oligomers and 11.3 g/L protein and this could support robust microbial growth. Marine wastes extract (MWE), prepared from marine organic wastes, and were used as nitrogen source for sulfate-reducing bacteria (SRB). These acidophiles occur in mine drainage and are exposed to high metal and sulfate concentrations (Dev and Bhattacharya, 2014). The MWE contains 13.95 g/L of nitrogen, and other micronutrients like K, Na, P, S, Ca, Fe, Mg, Mn, Zn, Co, Cu and Ni, and has a C/N ratio of 0.107. Protein pastes were prepared from shell waste through enzymatic treatment using trypsin, papain, pepsin and the average protein content in the protein paste was about 450 g/kg (Chakrabarti, 2002). Similarly, processing waste of prawns can be processed to protein powder mechanically or chemically with sodium hydroxide followed. Relative studies on the composition and amino acid profile, acceptability among consumers and nutritional quality of the protein powders revealed that the product prepared by freeze drying of the press liquor is superior in all aspects studied than the product prepared by mild alkali extraction.

Even though the substantial analyses and nutritional studies have demonstrated that the algal proteins are of high quality and comparable to conventional vegetable proteins, due to high production costs as well as technical hassles, the exploitation of algal protein is still in primary level (Becker, 2007). The use of whole microalgae biomass as a protein source in food and feed is simple and entrenched. However, there is an instant requirement for the development of practicable and robust processes, which can fractionate the microalgae biomass in different value-added products. Access to proteins from microalgae requires cell disruption and extraction. The critical review (Amorim et al., 2020) addresses the current state of the production of microalgae proteins for multifarious applications, and possibilities to concatenate the production of proteins and advanced biofuels.

Extraction of protein from agri-food residues, such as wheat bran, rice bran, brewer’s spent grains, pomegranate seeds, cauliflower leaves, pumpkin seed, and coffee beans has been widely studied (Liu et al., 2016; Phongthai et al., 2016; Xu et al., 2017; Talekar et al., 2018; Wen et al., 2020). During extraction, acid and alkali can hydrolyze proteins into smaller proteins or shorter peptides, which result in lower molecular weight of proteins in the extracts.

Hydrothermal liquefaction (HTL) of high moistured biomass such as microalgae, macroalgae, sludge, manure, and food waste, for the production of bio-oil has been widely concerned worldwide. A process for microalgae is based on extraction under high pressure, temperature and in supercritical water (Yang et al., 2018; Hietala et al., 2019). The high N content (20–40%) of these biomasses poses a challenge since up to 10% N may end up in the bio-oils may result as consequence of the HTL processing parameters (Leng et al., 2020). The nutrients present in the aqueous side fraction (Hu et al., 2017) can be reused in fermentation (Mehrabadi et al., 2016). The bio-oil obtained after HTL contains a large amount of nitrogen compared to that obtained by the solvent extraction method. On an average, total nitrogen (1–18 g/L), ammonium (0.34–12 g/L), and phosphate (0.7–12 g/L) are obtained (Shakya et al., 2017).

Pyrolysis of protein-rich biomass (e.g., algae, sewage sludge, lignocellulosic biomass) also suffer from a high nitrogen (N) content of the resulting bio-oils. The nitrogenous compounds present in these bio-oils are aliphatic and heterocyclic amines, amides and nitriles. Such bio-oil cannot be used as fuel directly since the high N content will induce massive emission of nitrogen oxides during combustion. The review by Leng et al. (2020) summarized the effects of biomass compositions and pyrolysis parameters on the contents of N and the *N*-containing chemical components in bio-oil.

### Outlook on Nitrogen-Containing Sidestreams

Due to the increase in global population, there is a need for new sources of protein and thus proteins from agri-food by-products have obtained increasing attention. Although growing, the animal feed market suffers from protein biorefinery by-products and recirculation of nitrogen has been identified as target to revamp the overall process. The newer protein conversion process developed will create value-added chemicals by recycling ammonia and releasing biofuels and high-value amino acids. Use of protein biomass in biogas production or by incineration to generate heat and electricity are limited. The use of agricultural and agro-based-industry wastes and seafood waste utilization will fabricate benefits in both environmental and economic aspects and using them as raw materials can help to diminish the overall production cost and will accord to the recycling of waste as well.

## Engineering *Corynebacterium glutamicum* for Access to Non-Native Substrates

**FIGURE 1:**
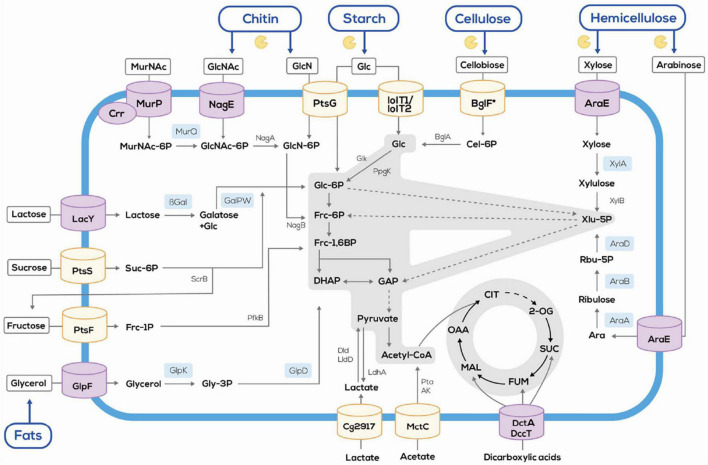
Schematic overview of the flexible feedstock concept established in *C. glutamicum* by systems metabolic engineering. Orchid cylinders and sky blue boxes indicate heterologous proteins. The symbol and blue lines indicate polysaccharides degradation *via* heterologous enzymes. AK, acetate kinase; Ara, arabinose; AraA, arabinose isomerase; AraB, ribulokinase; AraD, ribulose 5-phosphate 4-epimerase; AraE, arabinose transporter; βgal, β-galactosidase; BglA, phospho-β-glucosidase; BglF, cellobiose specific PTS; Cel-6-P, cellobiose-6-phosphate; Cg2917, putative L-lactate permease; Crr, glucose-specific PTS system enzyme IIA component; DccT, C4-dicarboxylate divalent anion/sodium symporter-type transporters; DHAP, dihydroxyacetone phosphate; Dld, quinone-dependent L-lactate dehydrogenase; Frc-1,6BP, fructose-1,6-bisphosphate; Frc-6P, fructose-6-phosphate; Frc-1P, fructose-1-phosphate; GalPW, galactose pathway; GAP, glyceraldehyde-3-phosphate; Glc-6P, glucose-6-phosphate; Glc, glucose; GlcN, glucosamine; GlcNAc, *N*-acetylglucosamine; GlcN-6P, glucosamine-6-phosphate, Glk, ATP dependent glucokinase; GlpD, glycerol-3-phosphate dehydrogenase; GlpF, glycerol facilitator; GlpK, glycerol kinase; Gly-3P, glycerol-3-phosphate; IolT1/IolT2, inositol transporters, also accepting glucose; LacY, lactose permease; LdhA, lactate dehydrogenase; LldD, quinone-dependent L-lactate dehydrogenase; MctC, monocarboxylic acid transporter; MurNAc, *N*-acetylmuramic acid; MurP, *N*-acetylmuramic acid PTS permease; MurQ, etherase; NagA, *N*-acetylglucosamine-6-P deacetylase, NagB, glucosamine-6-P deaminase; NagE, GlcNAc-specific PTS system from *Corynebacterium glycinophilum*; PfkB, 1-phosphofructokinase; PpgK, polyphosphate dependent glucokinase; Pta, phosphotransacetylase; PtsF, fructose-specific PTS; PtsG, glucose-specific PTS; PtsS, sucrose specific PTS; Rbu-5P, ribulose-5-phosphate; ScrB, sucrose-6-phosphate hydrolase; Suc-6P, sucrose-6-phosphate; Xlu-5P, xylulose-5-phosphate; XylA, xylose isomerase; XylB, xylulokinase.

Second generation feedstocks contain proteins and, thus, nitrogen. During biofuel production nitrogen is not used. However, it can be produced as by-product of biofuels (Huo et al., 2011). Alternatively, agri- and aquaculture sidestreams may be used for production of nitrogenous products such as amino acids, thus, incorporating the nitrogenous fraction of these second generation feedstocks. Several bacteria are used for fermentative production of amino acids, e.g., *E. coli* and *C. glutamicum*. One of the latest commercializations of an amino acid production process may be the *E. coli* process developed by Metabolic Explorer for fermentative production of L-methionine, which was acquired by Evonik (2019). For the use of alternative substrates, the industrially relevant *E. coli, C. glutamicum, Pseudomonas, Bacillus*, and yeast strains have been engineered to broaden their substrate scope (Wendisch et al., 2016). As compared to *E. coli*, the range of substrates available for *C. glutamicum* is narrow, e.g., including lactate, citrate, ethanol, and dicarboxylic acids besides glucose, fructose and sucrose (Polen et al., 2007; Arndt et al., 2008; Georgi et al., 2008; Youn et al., 2009). Metabolic engineering considerably widened the relatively narrow scope of the wild type (Wendisch and Lee, 2020). In the following, the metabolic engineering strategies to enable production of nitrogenous compounds from constituents of agri- and aquaculture sidestreams are detailed (Figure 1) and, where possible, examples for production of nitrogenous compounds by direct utilization of hydrolyzates are given.

### Engineering Access to Fats

Fats that are present in sidestreams may be transesterified with methanol or ethanol to yield the biodiesels FAME or FAEE, thus, they have an immediate biofuel application, while the stoichiometric byproduct glycerol accumulates. The *C. glutamicum* genes for glycerol kinase and glycerol-3-phosphate dehydrogenase are transcriptionally silent. Only upon overexpression of these genes or their respective orthologs from *E. coli* could the engineered strains grow and produce amino acids from glycerol (Rittmann et al., 2008; Meiswinkel et al., 2013b). The additional expression of the *E. coli* glycerol facilitator gene *glpF* increased the growth rate and volumetric productivity. Glycerol was used for production of the following nitrogenous compounds by recombinant *C. glutamicum*: amino acids L-glutamate, L-lysine, L-ornithine, L-arginine, and the diamine putrescine (Meiswinkel et al., 2013b).

### Engineering Access to Chitin

Chitin occurs in cell walls of fungi, the exoskeletons of crustaceans and insects, fish scales, radulae of mollusks, and cephalopod beaks, making it one of the most abundant biopolymers in nature. Technically, chitin is an accessible sidestream of marine fisheries (crustacean shells), mushroom farming and fungal fermentations for the production of enzymes (fungal mycelia). Chitin is made up of glucosamine and *N*-acetylglucosamine, whereas glucosamine and *N*-acetylmuramic acid constitute the bacterial cell wall peptidoglycan. The hexosamines serve as sources of carbon, energy and nitrogen, which is relevant in particular for production of nitrogenous compounds such as amino acids. Glucosamine is the only hexosamine utilized by wild-type *C. glutamicum*. It is taken up into the *C. glutamicum* cell and phosphorylated using phosphoenolpyruvate by a side activity of the glucose-specific phosphotransferase system PtsG (Uhde et al., 2013). Uptake and phosphorylation of *N*-acetylglucosamine to *N*-acetylglucosamine 6-phosphate required expression of the gene for the *N*-acetylglucosamine PTS (NagE) from *C. glycinophilum* (Matano et al., 2014). Since the native glucosamine 6-phosphate deaminase gene *nagB* is repressed by the transcriptional regulator NanR (Uhde et al., 2016), deletion of *nanR* or plasmid-borne overexpression of *nagB* efficient resulted in fast growth and production with *N*-acetylglucosamine (Matano et al., 2014; Uhde et al., 2016). *N*-Acetylmuramic acid is an *N*-acetylated sugar acid that makes up half of the polysaccharide content of peptidoglycan. When the *E. coli* genes *murP, crr*, and *murQ* coding for a PTS subunit specific for *N*-acetylmuramic acid, a general PTS component, and an etherase for cleavage of *N*-acetylmuramic acid 6-phosphate to yield *N*-acetylglucosamine 6-phosphate and lactate were expressed, *C. glutamicum* could utilize *N*-acetylmuramic acid (Sgobba et al., 2018a). L-Lysine was produced from glucosamine, *N*-acetylglucosamine as well as *N*-acetylmuramic acid (Matano et al., 2014; Uhde et al., 2016; Sgobba et al., 2018a).

A synthetic consortium composed of a chitin degrading *E. coli* strain and a *C. glutamicum* strain producing nitrogenous compounds has been used to develop chitin-based production (Vortmann et al., 2021). In this proof-of-concept study, the chitin monomer *N*-acetylglucosamine is hydrolyzed by a lysine-auxotrophic *E. coli* strain to yield acetate and glucosamine. Acetate is the only carbon source for the engineered *E. coli* strain, whereas the lysine-secreting *C. glutamicum* producer strain was engineered to only use glucosamine. Chitin degrading enzymes (chitinase, chitin deacetylase and glucosaminidase) had to be added to the growth media, but they may be synthesized and secreted by the *E. coli* strain used (Vortmann et al., 2021).

### Engineering Access to Seaweed

Seaweed has not been fully exploited as sustainable microbial feedstock for the fermentation industry. Algal biomass contains various polysaccharides, e.g., alginate and laminarin in brown macroalga that also contain mannitol. *C. glutamicum* cannot directly use the polymers, but possesses an arabitol utilization operon. The operon is repressed by the regulatory protein AtlR and can be induced by arabitol, but not by mannitol (Laslo et al., 2012). Strains lacking *atlR* utilize mannitol *via* arabitol uptake permease and arabitol dehydrogenase. This concept was taken further by genetic engineering inspired by carbon flux analysis leading to a titer of about 2 g/L L-lysine (Hoffmann et al., 2018). Combining systems metabolic engineering with heterologous fructokinase, a titer of about 76 g/L L-lysine was reached. Acid pre-treated seaweed *Durvillaea antarctica* or alginate-free extracts of the alga *Laminaria digitate* were sown to serve as carbon sources (Hoffmann et al., 2018). To make full use of macroalgal biomass *C. glutamicum* has to be engineered for growth with all major polysaccharide fractions present in brown, green and red algae.

### Engineering Access to Starch

Starch is not a native substrate of *C. glutamicum* wild type, but upon expression and secretion of α-amylase from *Streptomyces* L-lysine and L-glutamate were produced from starch (Seibold et al., 2006; Chen et al., 2019). In a concept similar to that described above for chitin, a synthetic *E. coli–C. glutamicum* consortium has been engineered for growth and production with starch (Sgobba et al., 2018b). The remaining challenges are that starchy biomass does have competing uses as food and feed, that debranching enzymes such as pullulanase have to be secreted or that starch has to be hydrolyzed to access cyclodextrin and branched starch portions (Burgardt et al., 2021).

### Engineering Access to Lignocellulosics

Lignocellulosic hydrolyzates such as spent sulfite liquor or pre-treated *Miscanthus* biomass have been used to produce nitrogenous compounds with metabolically engineered *C. glutamicum* strains (Joo et al., 2017; Sinner et al., 2021). Cellulose supported growth upon expression of exogenous genes for cellulases, β-glucanases, and β-glucosidases (Kim et al., 2014; Anusree et al., 2016). The hemicellulosic xylan fraction can be cleaved by endoxylanases and β-xylosidases to yield xylose (Imao et al., 2017). In the isomerase pathway that was transferred to *C. glutamicum*, xylose is converted to xylulose-5-phosphate, an intermediate of the pentose phosphate pathway, in a two-step pathway. While *C. glutamicum* possesses a gene for xylulose kinase, heterologous expression of a xylose isomerase was required. Combined overexpression of the genes coding for the respective enzymes allowed for growth (Kawaguchi et al., 2006), production of lysine and astaxanthin (Henke et al., 2018), L-ornithine (Eberhardt et al., 2014), 5-aminovalerate (Jorge et al., 2017b), and sarcosine (Mindt et al., 2019a). Transfer of the Weimberg pathway, which converts xylose to 2-oxoglutarate by five sequential enzyme reactions, supported growth with xylose when the exogenous *Caulobacter crescentus* genes were expressed (Radek et al., 2014). Later it was shown that it is sufficient to express either the *C. crescentus* genes encoding xylonate dehydratase or that coding for 2-oxo-3-deoxy-d-xylonate dehydratase (Brüsseler et al., 2019). Side reactions of two native enzymes (IolG and KsaD), a spontaneous lactonization reaction could be coupled to one of the two non-native dehydratases (either XylD or XylX) (Brüsseler et al., 2019). While *C. glutamicum* utilizes ribose, the other pentose in lignocellulosic hydrolyzates, arabinose, is not a native substrate, but overexpression of the *araBAD* operon from *E. coli* led to production of L-glutamate, L-lysine, L-ornithine, and L-arginine, respectively, from arabinose (Schneider et al., 2011). Rice straw hydrolyzates supported amino acid production best when both *araBAD* and *xylAB* were overexpressed (Meiswinkel et al., 2013a). The fact that *C. glutamicum* readily utilizes acetate as sole and combined source of carbon and energy (Gerstmeir et al., 2003) was exploited for production using blends, e.g., of acetate and xylose (Mindt et al., 2019b) or of lignocellulosic acetate (Kiefer et al., 2021).

### Engineering Access to Methanol and Monomethylamine

*Corynebacterium glutamicum* cannot utilize carbon sources that lack carbon–carbon bonds such as methanol. Methanol can be obtained by reducing carbon dioxide and is considered a valuable carbon source for biotechnological processes (Clomburg et al., 2017). The knowledge about natural methylotrophs that can grow fast and efficiently with methanol (Müller et al., 2015) inspired metabolic engineering of *C. glutamicum* for methanol utilization. Naturally, *C. glutamicum* oxidizes methanol to carbon dioxide yielding reduction equivalents. To assimilate methanol into biomass or products such as L-lysine and cadaverine ribulose monophosphate cycle genes from natural methylotrophs had to be expressed in *C. glutamicum* (Lessmeier et al., 2015). This was demonstrated for production of L-lysine and cadaverine in an isotope-labeling experiment (Lessmeier et al., 2015). Methanol-essential growth was achieved for *E. coli* (Meyer et al., 2018) and for glutamate-producing *C. glutamicum* (Tuyishime et al., 2018). Adaptive laboratory evolution led to higher recalcitrance to methanol and helped to identify bottlenecks such as formation of methanethiol as toxic by-product (Hennig et al., 2020; Wang et al., 2020).

Monomethylamine is synthesized chemically from ammonia and methanol, but it also occurs in nature, e.g., due to putrefaction of proteins or by demethylation of methylated L-arginine and L-lysine residues present in histone proteins. Some methylotrophs are able to utilize monomethylamine as sole source of energy, carbon and nitrogen, but *C. glutamicum* cannot use monomethylamine as growth substrate. However, monomethylamine may serve as co-substrate for the production of *N*-methylated amines and amino acids by recombinant *C. glutamicum* strains. In these strains, reductive methylamination of a 2-oxoacid by a side activity of the *Pseudomonas putida* enzyme DpkA (Muramatsu et al., 2005), which in this bacterium reduces Δ1-piperideine-2-carboxylate to L-pipecolic acid, yields *N*-methylated amino acids, e.g., pyruvate can be reductively aminated by DpkA using monomethylamine to *N*-methyl-L-alanine (Mindt et al., 2018). This concept was also applied to reductive methylamination of glyoxylate and phenylpyruvate to yield sarcosine (*N*-methyl-glycine) and *N*-methyl-L-phenylpyruvate, respectively (Mindt et al., 2020; Kerbs et al., 2021).

### Outlook on Engineering *Corynebacterium glutamicum* for Utilization of Non-native Substrates

One aspect of metabolically engineered strains relevant for large-scale fermentation processes is genetic stability. The metabolic engineering approaches listed in Table 1 comprise both chromosomal modifications and plasmid-borne gene expression. The more common rolling circle-replicating plasmids are less stabile than theta-type replicating plasmids and shifting from rolling circle-replicating to theta-type replicating plasmids may improve productivity as shown, e.g., for production of the diamine cadaverine by *B. methanolicus* (Irla et al., 2016). However, regarding genetic stability, chromosomal changes introduced into the genome of the production host remains the gold standard of genetic engineering.

**TABLE 1:** Summary of fermentative production of nitrogenous compounds by engineered *C. glutamicum* from renewable carbon sources.

Product	Carbon source	Genotype or strain	Plasmid-based expression	Titer (g/L)	Productivity (g/L/h)	Cultivation	References
γ-Aminobutyrate	Glucose	Δ*argB:gad (Lactobacillus plantarum*) Δ*proB:gad*Δ*dapA:plk (L. plantarum*)		70.6	1.04	Fed-batch	Zhang et al., 2019
	Glucose	Δ*gapP*Δ*gapT*	Secretory expression of *gadB*^m^* (E. coli)*	77.6	1.21	Fed-batch	Wen and Bao, 2021
	Glucose and xylose from empty fruit bunch solution	L-Glutamate producer KCTC 1447	*gadB*^Glu89Gln/Δ 452–466^ (*E. coli*)-*xylAB* (*E. coli*)	35.5		Batch	Baritugo et al., 2018
	Glucose	*odhA*^TTG^ *odhI*^T15A^ Δ*argF*Δ*argR*Δ*snaA*Δ*DgabTDP* Δ*yggB*Δ*cgmA*	*patDA* (*E. coli*), *speC* (*E. coli*)-*gapA*-*pyc*-*argB*^A49V,M54V^-*argF*_21_	63.2	1.34	Fed-batch	Jorge et al., 2017a
	Glucose Xylose Glucosamine *N*-Acetylglucosamine	Δ*nanR* Δ*nanR*	*xylA* (*Xanthomonas campestris*) *nagE* (*C. glycinophilum*)	3.3 1.2 1.8 3.3		Flask, 100 mM respective carbon source	
Putrescine	Glucose	Δ*argRF odhA*^TTG^ *odhI*^T15A^ Δ*snaA*	*speC* (*E. coli*)-*argF*_21_-*gapA*-*pyc*-*argB*^A49V,M54V^	5.1	0.21	Flask	Nguyen et al., 2015
	Crude glycerol	Δ*argF*Δ*argR*	*speC* (*E. coli*)-*argF*_21_, *glpFKD* (*E. coli*)	0.5		Flask, 20 g/L crude glycerol RG1	Meiswinkel et al., 2013b
	Xylose	Δ*argF*Δ*argR*	*speC* (*E. coli)*-*argF*_21_, *xylA* (*X. campestris)-xylB* (*C. glutamicum*)	2.5		Flask, 100 mM xylose	Meiswinkel et al., 2013a
5-Aminolevulinic acid	Glucose	Δ*sucCD*	*hemA* (*Rhodobacter capsulatus*)-*rhtA* (*E. coli*)	14.7	0.92	Flask, 10 g/L glycine	Yang et al., 2016
	Cassava bagasse hydrolyzate Corn starch hydrolyzate		*hemA* (*R. palustris*)-*ppc*	18.5 16	0.47 0.42	Fed-batch, glycine addition	Chen et al., 2020
*N*-Methyl-L-alanine	Glucose	Δ*aceE*Δ*pqo*Δ*ldhA*Δ*C-T ilvN*Δ*alaT*Δ*avtA*	*dpkA* (*Pseudomonas putida*)	31.7	0.35	Fed-batch, monomethylamine (MMA) and acetate addition	Mindt et al., 2018
	Starch Arabinose Xylose		*amyA* (*Streptomyces griseus*) *araBAD* (*E. coli*) *xylA* (*X. campestris*)-*xylB* (*C. glutamicum*)	7.5 4.2 7.0		Flask, 30 g/L respective C-source, 16.6 g/L K-acetate, and 9.3 g/L MMA	
Sarcosine	– Glucose Arabinose	Δ*aceB icd*^GTG^ – –	*dpkA* (*P. putida*) – *araBAD* (*E. coli*)	2.4 3.4		Flask, 12 g/L respective C-source, 20 g/L K-acetate, 3.1 g/L MMA	Mindt et al., 2019b
	Xylose	–	*xylA* (*X. campestris*)-*xylB* (*C. glutamicum*)	8.7	0.12	Flask, 12 g/L respective C-source, 20 g/L K-acetate, 6.2 g/L MMA	
Ectoine	Glucose and molasses	*lysC*^T311I^ Δ*lysE*Δ*ddh:P_*tuf*_ectABCD* (*P. stutzeri*)	*ectABC*^opt^ (*P. stutzeri*)	65	1.16	Fed-batch	Giesselmann et al., 2019
	Glucose	*pyc*^P458S^ *lysC*^T311I^ *hom*^V59A^ Δ*sugR* Δ*ldhA*	*ectABC* (*Chromohalobacter salexigens*)	22	0.32	Fed-batch	Pérez-García et al., 2017b
	Glucose Glycerol Glucosamine Xylose Arabinose Soluble starch (+ Glu 2.5 g/L)	–	*glpKDF* (*E. coli*) *nagB* (*C. glutamicum*) *xylA* (*X. campestris*)-*xylB* (*C. glutamicum*) *araBAD* (*E. coli*) *amyA* (*S. griseus*)	0.7 0.6 0.8 0.4 0.4 0.5	– 0.034 0.045 – – –	Flask, 10 g/L respective C-source	
3-Amino-4-hydroxybenzoic acid	Sweet sorghum juice	L-lysine-producer ATCC 21799	*griH-griI* (*Streptomyces griseus*)	1.0		Flask	Kawaguchi et al., 2015
L-Pipecolic acid	Glucose and sucrose	L-Lysine producer GRLys1 Δ*sugR*Δ*ldhA*Δ*lysE*^2^	*lysDH* (*Silicibacter pomeroyi*)-*proC*	14.4	0.21	Fed-batch	Pérez-García et al., 2017a
	Glycerol Xylose Glucosamine Soluble starch		*glpKDF* (*E. coli*) *xylA* (*X. campestris*)-*xylB* (*C. glutamicum*) *nagB* (*C. glutamicum*) *amyA* (*S. griseus*)	1.42 0.52 0.34 1.55		Flask, 10 g/L respective C-source (+glu 2.5 g/L)	
Cadaverine	Glucose	L-Lysine producer LYS-12 Δ*bioD:*P*_*tuf*_ldcC*^opt^ (*E. coli*) Δ*NCgl1469* Δ*lysE* P*_*sod*_cg2893*		88	2.2	Fed-batch	Kind et al., 2014
	Glucose	L-Lysine producer PKC Δ*lysE:H30-ldcC* (*E. coli*)		103	1.47	Fed-batch	Kim et al., 2018
	Xylose	Cadaverine producer DAP-Xyl1 *icd*^GTG^ *P*_eftu_*fbp* P*_*sod*_tkt*Δ*act*Δ*lysE*		103	1.5	Fed-batch	Buschke et al., 2013
5-Aminovalerate	Glucose *Miscanthus* hydrolyzate solution	L-Lysine producer KCTC 1857	*davBA* (*P. putida*)	39.9 12.5	0.54 0.40	Fed-batch	Joo et al., 2017
	Glucose	L-Lysine producer GRLys1 Δ*sugR*Δ*ldhA*Δ*snaA*Δ*cgmA*Δ*gabTDP*	*ldcC* (*E. coli*), *patDA* (*E. coli*)	5.1	0.12	Flask	Jorge et al., 2017b; Sasikumar et al., 2021
	Glucosamine Arabinose Starch (+ Glu 2.5 g/L) Xylose Rice straw hydrolyzate		*nagB* (*C. glutamicum*) *araBAD* (*E. coli*) *amyA* (S. *griseus*) *xylA* (*X. campestris*)-*xylB* (*C. glutamicum*) *xylA* (*X. campestris*)-*xylB* (*C. glutamicum*)	1.22 0.26		Flask, 10 g/L respective C-source Glu 24 g/L + Xyl 14 g/L	
7-Chloro-L-tryptophan	Glucose	Δ*csm*Δ*trpL:*P*_*ilvCM*1_ trpE*^fbr^ Δ*vdh:P_*ilvC*_aroG*^fbr^	*trpD* (*E. coli*), *rebH*-*rebF* (*Lechevalieria aerocolonigenes*)	0.11			Veldmann et al., 2019b
	Arabinose Glucosamine Xylose		*araBAD* (*E. coli*) *nagB* (*C. glutamicum*) *xylA* (*X. campestris*)-*xylB* (*C. glutamicum*)	0.052 0.051 0.034		Flask, 40 g/L respective C-source	
*N*-Methyl-L-phenylalanine	Glucose	Anthranilate producer ARO9 Δ*trpEG*Δ*ilvE* Δ*aroT*	*dpkA*^P262A,M141L^ (*P. putida*), *pheA*^FBR^ (*E. coli*)	0.73	0.01	Flask, 20 g/L glucose, 0.35 M MMA	Kerbs et al., 2021
	Xylose		*xylA* (*X. campestris*)-*xylB* (*C. glutamicum*)	0.6	0.008	12 g/L xylose, 0.35 M MMA	

Notably, all *C. glutamicum* strains rely on ammonium salts or urea to provide nitrogen for sidestream-based production of nitrogenous compounds. This is due to the fact that the bacterium does not secrete proteases or peptidases. Only access to amino sugars has been established for *C. glutamicum* using exogenous genes. Monomethylamine (s. above) that is present in aquaculture sidestreams can be used for production *N*-alkylated amino acids by *C. glutamicum* strains that use it to reductively aminate 2-oxo acids. Thus, we predict that future metabolic engineering of *C. glutamicum* may include secretion of proteases and peptidases to access proteins and peptides and other polymeric nitrogen-containing substrates as nitrogen sources, thus, enlarging the currently available nitrogen substrate scope (ammonium, urea, some amino sugars, some alkylamines).

## Extending and Intercepting Biosynthetic Routes to Produce Nitrogenous Compounds

**FIGURE 2:**
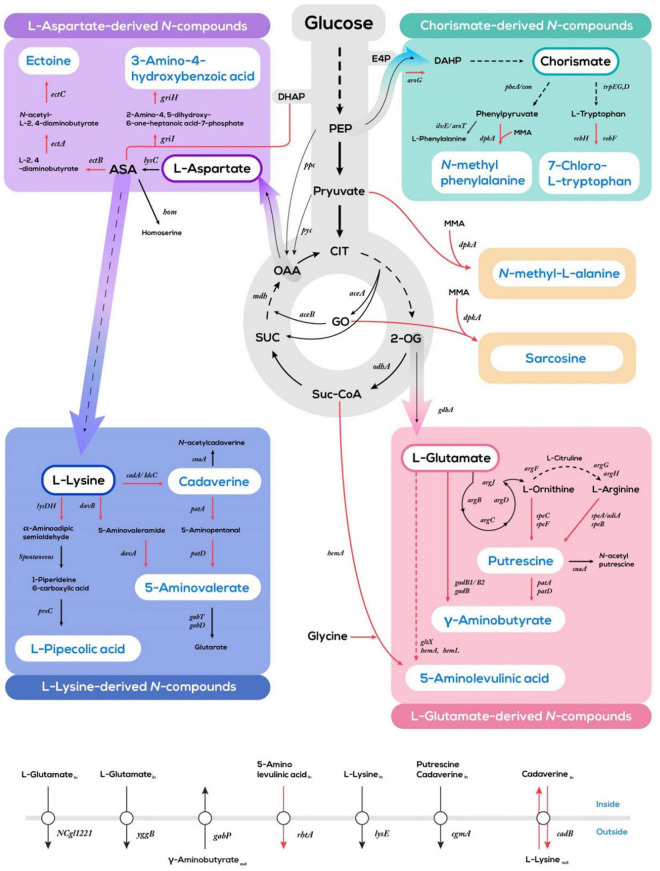
Schematic overview of systems metabolic engineering of *C. glutamicum* for the production of nitrogenous compounds from glucose. Red lines indicate artificial synthetic pathways; black colored lines indicate endogenous pathways. Multi-step reactions are represented by dotted lines. E4P, erythrose 4-phosphate; DHAP, dihydroxyacetone phosphate; DAHP, 3-deoxy-D-arabinoheptulosonate 7-phosphate; PEP, phosphoenolpyruvate; CIT, citrate; 2-OG, 2-oxoglutarate; Suc-CoA, succinyl-CoA; SUC, succinate; OAA, oxaloacetate; GO, glyoxylate; ASA, L-aspartate 4-semialdehyde; MMA, monomethylamine.

Diverse nitrogenous compounds have been produced by systems metabolic engineering of *C. glutamicum* through extending and intercepting metabolic pathways derived from glycolysis, TCA cycle, aspartate-family pathways, and the shikimate pathway (Figure 2). The combined metabolic networks of reconstituted synthetic pathways with utilization pathways for alternative carbon sources have accelerated sustainable production of nitrogenous compounds from agri- and aqua-culture sidestreams (Baritugo et al., 2018; Wolf et al., 2021). Here, the product portfolio accessible *via C. glutamicum* fermentation primarily features amino acids, halogenated amino acids, diamines, and alkylamines (Wendisch and Lee, 2020; Table 1). Besides, examples of the design and implementation of synthetic pathways not present in native metabolism to produce sought after nitrogenous compounds will be provided.

### Central Metabolic Pathway-Derived Nitrogenous Compounds

*Corynebacterium glutamicum* traditionally employed for commercial production of L-glutamate is an ideal platform strain for the production of various nitrogenous compounds derived from L-glutamate, including γ-aminobutyrate (Takahashi et al., 2012), L-ornithine (Jensen and Wendisch, 2013; Jensen et al., 2015), L-arginine (Jensen et al., 2015), putrescine (Schneider et al., 2012), and 5-aminolevulinic acid (Ramzi et al., 2015; Figure 2 and Table 1). Additionally, the intermediates in glycolysis and TCA cycle can be converted to nitrogenous compounds including 5-aminolevulinate (Yang et al., 2016), *N*-methyl-L-alanine (Mindt et al., 2018), and sarcosine (Mindt et al., 2019b) by introducing synthetic pathways and/or intercepting unnecessary pathways into *C. glutamicum*.

#### γ-Aminobutyrate

γ-Aminobutyrate (GABA), one of the non-proteinogenic ω-amino acids, functions as a primary inhibitory neurotransmitter in animals and has several therapeutic activities, including anti-anxiety, anti-depression, anti-schizophrenia, analgesics, diuresis, and hypotension (Zhang et al., 2014; Shi et al., 2017). In addition, butyrolactam is derived from GABA by ring closure and is used to synthesize the biodegradable polyamide (PA, nylon) PA 4 by ring-opening condensation (Baritugo et al., 2018).

As the conversion of L-glutamate to GABA is mediated by pyridoxal phosphate (PLP)-dependent glutamate decarboxylase (GAD; EC 4.1.1.15), *C. glutamicum* with a superior L-glutamate pool has been employed for efficient GABA production from renewable sources. Pioneering studies for GABA production using *C. glutamicum* were performed by heterologous expression of *gadB* from *E. coli* (Takahashi et al., 2012), *gadB1*/*gadB2* from *Lactobacillus brevis* (Shi and Li, 2011), and *gad* from *Lactobacillus plantarum* (Zhang et al., 2014). Improving GAD activities from *L. brevis* and *E. coli* with broadening active pH range to neutral was obtained by protein engineering, yielding increase of GABA production in *C. glutamicum* (Choi et al., 2015). With these features, Baritugo et al. (2018) achieved 35.5 g/L of GABA at bath fermentation using empty fruit bunch solution containing glucose and xylose by recombinant *C. glutamicum*, in which *E. coli gadB*^m (Glu89Gln/Δ452–466)^ and *xylAB* genes encoding xylose isomerase and xylulokinase were expressed under control of the promoter H36. Due to the requirement of PLP as a cofactor of GAD and absence of pyridoxal kinase in *C. glutamicum*, an engineered strain was accomplished by expression of *plk* encoding pyridoxal kinase and *gad* from *L. plantarum*, together with blocking of byproducts formation pathways (Zhang et al., 2014). In fed-batch cultures, 70.6 g/L of GABA were accumulated using glucose after 70 h *via* a two-stage pH control. Increase of intracellular pool of L-glutamate for GABA production was attained by deletion of *pknG* encoding serine/threonine protein kinase (Okai et al., 2014), overexpression of *ppc* or deletion of *mdh* (Shi et al., 2017), or deletion of *NCgl1221* encoding L-glutamate exporter (Cho et al., 2017). Another approach to enhance GABA production was conducted by secretion of GAD enzyme and blocking GABA import and degradation (Δ*gapP*Δ*gapT*), whereby extracellular accumulated L-glutamate was directly converted to GABA (Wen and Bao, 2021). Consequently, 77.6 g/L of GABA with a yield of 0.44 g/g were accumulated on fed-batch fermentation. A new route for GABA production *via* the growth-associated conversion of putrescine to GABA was established by heterologous expression of *patA* coding for putrescine transaminase and *patD* coding for γ-aminobutyraldehyde dehydrogenase from *E. coli* (Jorge et al., 2016). Improved production of GABA was taken by blocking by-product formation of *N*-acetylputrescine (Δ*snaA*), avoiding export of L-glutamate and putrescine (Δ*yggB*Δ*cgmA*), and increasing precursor supply by reducing 2-oxoglutarate dehydrogenase activity (*odhA*^TTG^*odhI*^T15A^) (Jorge et al., 2017a). The engineered strain enabled production of 63.2 g/L of GABA with the highest volumetric productivity up to 1.34 g/L/h. Moreover, expression of *xylA* from *Xanthomonas campestris*, deletion of *nanR*, and deletion of *nanR* and expression of *nagE* from *Corynebacterium glycinophilum*, respectively, led to production of GABA using sustainable resources xylose, glucosamine, and *N*-acetyl-glucosamine. Of these, GABA titer and productivity from *N*-acetyl-glucosamine were higher than those from D-glucose.

#### Putrescine

Polyamides are polymers in which the repeating building blocks, amine and carboxylic acid or lactams, are linked by amide bonds (Kind et al., 2010). Commercially available PA 6 and PA 6,10 are typically produced *via* chemical routes from petroleum-based chemicals. Due to shortage of fossil resources and environmental concerns, bio-based production of polyamides using sustainable resources has attracted considerable interest. Putrescine (1,4-diaminobutane) is a 4-carbon aliphatic diamine and a commodity building block for the synthesis of bio-based PA 4,6 and PA 4,10 (Baritugo et al., 2018).

*Corynebacterium glutamicum* has a competitive potential for putrescine production due to its innate abilities, including overproduction of the main precursor L-glutamate, remarkable tolerance to high-concentrations of putrescine, and no putrescine degradation routes (Schneider and Wendisch, 2010). Putrescine production by *C. glutamicum* was first evaluated by recruiting two alternative routes *via* the ODC (ornithine decarboxylase) and ADC (arginine decarboxylase) pathways from *E. coli* (Schneider and Wendisch, 2010). As a result, the putrescine yield in *C. glutamicum* implanted the ODC pathway was 40-fold higher than that provided with the ADC pathway. Putrescine production was further enhanced by following strategies: (i) inactivation of L-arginine repressor by deletion of *argR*, (ii) fine-tuning *argF* expression encoding ornithine transcarbamoylase through modulation of transcription and translation efficiencies, (iii) reducing 2-oxoglutarate dehydrogenase activity by chromosomal changes *odhA*^TTG^ and *odhI*^T15A^, (iv) increasing precursor supply flux by expression of *gapA* and *pyc* genes, (v) deregulation of ArgB by introducing mutated *argB* encoding feedback-resistant *N*-acetylglutamate kinase, (vi) blocking accumulation of by-product *N*-acetylputrescine by deletion of *snaA* (Nguyen et al., 2015). Expression of *speC* from *E. coli* in engineered *C. glutamicum* harboring genetic properties described above led to production of 5.1 g/L putrescine with a productivity of 0.21 g/L/h in baffled flasks. Another strategy to improve putrescine production was first attempted by construction of ornithine overproducing strain, followed by plasmid-mediated expression of *speC* from *E. coli* resulted in production of putrescine with a yield on glucose of 0.17 g/g (Jensen et al., 2015). Putrescine production from crude glycerol (Meiswinkel et al., 2013b), hemicellulosic xylose (Meiswinkel et al., 2013a), and glucosamine (Uhde et al., 2013) as alternative carbon sources has been demonstrated by expression of *glpFKD* from *E. coli*, *xylA* from *X. campestris* and *xylB* from *C. glutamicum*, and *nagB* from *C. glutamicum*, respectively, in engineered strains.

#### 5-Aminolevulinic Acid

5-Aminolevulinic acid (5-ALA) is a 5-carbon non-proteinogenic δ-amino acid and is a major precursor of tetrapyrroles, including porphyrin, heme, chlorophyll, and vitamin B_12_ (Chen et al., 2020). 5-ALA can be used in medicine, agriculture, and feed additive as photodynamic therapy, a potential bio-stimulant for crop production, and a feed amino acid for livestock rearing.

Two kinds of the artificial pathways were introduced for 5-ALA production in *C. glutamicum*. The C5 pathway comprised three enzymatic steps converting L-glutamate to 5-ALA *via* L-glutamyl-tRNA and L-glutamate-1-semialdehyde, and corresponding enzymes are glutamyl-tRNA synthetase, glutamyl-tRNA reductase, and glutamate-1-semialdehyde aminotransferase encoded by *gltX*, *hemA*, and *hemL* (Ramzi et al., 2015). 5-ALA-producing strain has been engineered by expression of a mutated *hemA* from *Salmonella typhimurium* and *hemL* from *E. coli*, together with flux redistribution of TCA cycle by mutation of OdhI^T14A/T15A^ and introduction of threonine/homoserine exporter RhtA from *E. coli* (Ramzi et al., 2015; Ko et al., 2019). Under ethambutol induction condition, 5-ALA production reached to 2.9 g/L in shaking incubator. The C4 pathway was reconstituted by expression of different sources of *hemA* encoding 5-aminolevulinic acid synthase by which succinyl-CoA and glycine are condensed to form 5-ALA (Feng et al., 2016; Yang et al., 2016; Chen et al., 2020). Of these, expression of *hemA* from *Rhodobacter capsulatus* and *rhtA* encoding threonine/homoserine exporter from *E. coli* in *C. glutamicum* (Δ*sucCD*) showed the highest titer of 5-ALA (14.7 g/L) from D-glucose and glycine in fed-batch fermentation (Yang et al., 2016). Similarly, optimized expression of *hemA* from *R. palustris* as well as additional expression of *ppc* gene led to 16.3 g/L of 5-ALA from D-glucose and glycine (Chen et al., 2020). Use of cassava bagasse hydrolyzate and corn starch hydrolyzate as cheap raw materials resulted in accumulation of 18.5 g/L and 16 g/L of 5-ALA, respectively, whereas use of beet molasses gave rise to serious suppression of 5-ALA production.

#### *N-*Methyl-L-Alanine

*N*-Methylated amino acids are important building blocks for peptide-based drugs such as vancomycin, actinomycin D, cyclosporine A, and *N*-methylated tubulysin and received increasing interest in the pharmaceutical industry due to improved pharmacokinetic properties (Mindt et al., 2018; Wendisch and Lee, 2020). Since chemical syntheses of *N*-methylated amino acids are often limited by low yield, incomplete stereoselectivity, and side reactions, microbial and biocatalytic approaches for their production may offer cleaner and greener alternatives over chemical methods (Muramatsu et al., 2005; Mindt et al., 2020).

Typically, *N*-methyl amino acid dehydrogenase/imine reductase DpkA from *P. putida* can catalyze the reductive alkylamination of 2-oxo acids to yield the corresponding *N*-alkylamino acids (Mihara et al., 2005). As a proof-of-concept, fermentative production of *N*-methyl-L-alanine (NMeAla), one of representative *N*-alkylamino acids, was obtained by introduction of *dpkA* from *P. putida* in pyruvate-overproducing *C. glutamicum*, which generated 31.7 g/L of NMeAla from D-glucose and monomethylamine (MMA) in fed-batch fermentation (Mindt et al., 2018). In addition, sustainable production of NMeAla from alternative feedstocks was achieved by extending metabolic pathways to utilize starch, arabinose, or xylose.

#### Sarcosine

Sarcosine (*N*-methylglycine) is ubiquitous in biological materials and occurs as an intermediate in choline and glycine metabolism (Mindt et al., 2019b). It is used in cosmetic formulations such as hair conditioners and surfactant cleansers and has clinical significance for the treatment of schizophrenia and depression (Hyslop et al., 2019).

Due to its catalytic efficiency and broad substrate specificity, DpkA from *P. putida* is able to catalyze the formation of sarcosine from glyoxylate as substrate for *N*-alkylation in the presence of MMA. Accumulation of glyoxylate in *C. glutamicum* resulted from deletion of *aceB* encoding malate synthase and the start codon exchange of gene *icd*^GTG^ encoding isocitrate dehydrogenase (Mindt et al., 2019b). Then optimized expression of *dpkA* in the constructed strains enabled production of 2.4 g/L, 2.7 g/L, and 3.4 g/L of sarcosine, respectively, from glucose, lignocellulosic xylose and arabinose supplemented with potassium acetate. Besides, 8.7 g/L of sarcosine with a productivity of 0.12 g/L/h were achieved in a xylose-based optimized culture, demonstrating that sarcosine production from xylose and arabinose outperforms glucose-based production. The mutation of DpkA (DpkA^F117L^) in the substrate binding site led to increased specific activity toward glyoxylate and MMA as substrates and allowed faster production of sarcosine (0.16 g/L/h) compared to the control enzyme from xylose-based fermentation process (Mindt et al., 2019b).

### Aspartate Pathway-Derived Nitrogenous Compounds

In engineered *C. glutamicum* strains, the coupled metabolic networks for L-lysine biosynthesis *via* L-aspartate and utilization of several alternative carbon sources have been extended not only by synthetic pathways *via* L-aspartate 4-semialdehyde for production of ectoine (Becker et al., 2013) and 3-amino-4-hydroxybenzoic acid (Kawaguchi et al., 2015), but also L-lysine degradation pathways for production of L-lysine-derived nitrogenous compounds, such as L-pipecolic acid (Pérez-García et al., 2016, 2019), cadaverine (Kim et al., 2018), and 5-aminovalerate (Rohles et al., 2016; Figure 2 and Table 1).

#### Ectoine

The cyclic amino acid ectoine (1,4,5,6-tetrahydro-2-methyl-4-pyrimidinecarboxylic acid) is a representative compatible solute that is wide-spread among halophilic and halotolerant eubacteria (Liu et al., 2021). It not only functions as osmoprotectant, biofunctional stabilizer, skin protector, and potential drugs for diseases but also is widely used in cosmetic and medical industries (Liu et al., 2021). Since ectoine production in the halophilic microbes through ‘bacterial milking’ process causes corrosive damage to devices, its production process under low salt conditions is crucial for economical production (Wolf et al., 2021). In this sense, *C. glutamicum* has emerged as a prominent strain for ectoine production.

The biosynthesis of ectoine from L-aspartate 4-semialdehyde involves three enzymes, namely, L-2,4-diaminobutyrate aminotransferase (EctB), L-2,4-diaminobutyrate acetyltransferase (EctA), and ectoine synthase (EctC). The recombinant strain for ectoine production was implemented by expression of codon-optimized *ectABCD* from *Pseudomonas stutzeri* in a strain with *lysC*^T311I^ mutation followed by inactivation of L-lysine exporter (Becker et al., 2013). The resulting strain yielded 4.5 g/L of ectoine under low-salt conditions after 16 h cultivation. Subsequently, *C. glutamicum* that contained an optimized heterologous ectoine pathway with a fine-tuned monocistronic expression of *ectB*, *ectA*, and *ectC* genes produced 65 g/L ectoine on a glucose-molasses medium within 56 h in a fed-batch process (Giesselmann et al., 2019). On the other hand, Pérez-García et al. (2017b) developed an ectoine-producing *C. glutamicum* from L-lysine-producing basal strain (*pyc*^P458S^ *lysC*^T311I^ *hom*^V59A^), because L-lysine and ectoine share L-aspartate 4-semialdehyde as a same precursor. The strategies comprised expression of *ectABC* from *Chromohalobacter salexigens*, deletion of *sugR* encoding DeoR-type regulator, and deletion of *ldhA* encoding lactate dehydrogenase. 22 g/L of ectoine and 0.32 g/L/h of productivity were obtained in a fed-batch culture. Additionally, *C. glutamicum* strains utilizing the several carbon sources, i.e., glycerol, glucosamine, xylose, arabinose, and soluble starch, were achieved by introduction of *glpKDF* from *E. coli*, *nagB* from *C. glutamicum*, *xylA* from *X. campestris* and *xylB* from *C. glutamicum*, *araBAD* from *E. coli*, and *amyA* from *Streptomyces griseus*, respectively. Among tested alternatives carbon sources, the highest ectoine yield was obtained using glucosamine, providing both carbon and nitrogen sources for *C. glutamicum*.

#### 3-Amino-4-Hydroxybenzoic Acid

3-Amino-4-hydroxybenzoic acid (3,4-AHBA) is a benzene derivative that acts as a precursor for a variety of secondary metabolites produced by *Streptomyces* and for the synthesis of polybenzoxazole, a thermostable bioplastic (Suzuki et al., 2006; Kawaguchi et al., 2015). Unlike most aromatic compounds synthesized *via* the shikimate pathway, it is formed from L-aspartate 4-semialdehyde and dihydroxyacetone phosphate by the activities of GriI and GriH. A 3,4-AHBA-producing *C. glutamicum* was derived from L-lysine-producing *C. glutamicum* by transformation of a plasmid expressing *griH* and *griI* genes from *S. griseus* (Kawaguchi et al., 2015). Owing to fermentative properties of *C. glutamicum* utilizing sucrose, the engineered strain led to accumulation of 1.0 g/L 3,4-AHBA from sweet sorghum juice, an alternative feedstock for sugar cane molasses, which harbors fermentable sugars and amino acids as a nitrogen source. Moreover, this novel pathway has guided an alternative route for the biosynthesis of a benzene ring from C_3_ and C_4_ metabolites independent of the shikimate pathway.

#### L-Pipecolic Acid

The cyclic α-amino acid L-pipecolic acid (piperidine 2-carboxylic acid, L-PA) is a critical regulator of inducible plant immunity and an important precursor of immunosuppressants, antitumor agent, or peptide antibiotic (Pérez-García et al., 2016, 2019).

The production of L-pipecolic acid *via* L-lysine was attempted by introduction of *lysDH* encoding lysine 6-dehydrogenase from *Silicibacter pomeroyi* combined with endogenous expression of *proC* encoding pyrroline 5-carboxylate reductase in the L-lysine-producing *C. glutamicum* (GRLys1Δ*sugR*Δ*ldhA*) (Pérez-García et al., 2016). This allowed production of L-PA to a titer of 1.8 g/L with a productivity 0.04 g/L/h. Transport engineering of *C. glutamicum* by abolishing L-lysine export resulted in production of 3.9 g/L of L-PA without accumulation of L-lysine in shake flask cultivation (Pérez-García et al., 2017a). The resulting strain produced 14.4 g/L of L-PA with a productivity 0.21 g/L/h by fed-batch cultivation using glucose and sucrose as carbon sources. Finally, heterologous expression of relevant genes (described above) for utilization of alternative carbon sources, i.e., glycerol, xylose, glucosamine, and soluble starch, resulted in production of L-PA from these sustainable resources.

#### Cadaverine

Cadaverine (1,5-diaminopentane) is a 5-carbon aliphatic diamine and can be used as a platform chemical for the synthesis of bio-polyamide PA 5,4 and PA 5,10 (Buschke et al., 2013).

Cadaverine is formed from L-lysine catalyzed by lysine decarboxylases (E.C. 4.1.1.18). The production of cadaverine was accomplished in *C. glutamicum* with a high intracellular L-lysine pool through extending L-lysine pathway by optimal expression of several sources of lysine decarboxylase genes, including *cadA* and *ldcC* from *E. coli* and *ldc* from *Hafnia alvei* (Mimitsuka et al., 2007; Kind et al., 2010; Li et al., 2014; Oh et al., 2015). Improvement of cadaverine production was implemented with the following strategies. These include blocking the accumulation of acetylated cadaverine (Δ*snaA*) (Nguyen et al., 2015), together with engineering of cellular transport processes, such as deletion of L-lysine exporter (*lysE*), overexpression of a putative putrescine and cadaverine exporter gene (*cgmA*, *cg2893*) (Kind et al., 2011; Lubitz et al., 2016), and overexpression of *cadB* encoding cadaverine-lysine antiporter from *E. coli* (Li et al., 2014). The breakthrough works to boost cadaverine yield were done from L-lysine-overproducing strains in accordance with the strategies described above by Kind et al. (2014) and Kim et al. (2018). The resulting strains, respectively, allowed titers of 88 g/L and 103 g/L of cadaverine with productivities of 2.2 g/L/h and 1.47 g/L/h in fed-batch processes. The sustainable production of cadaverine in *C. glutamicum* was performed by using alternative renewable sources, e.g., soluble starch (Tateno et al., 2009), the hemicellulose sugar xylose (Buschke et al., 2011), xylooligosaccharides (Imao et al., 2017), methanol (Lessmeier et al., 2015), and cellobiose (Ikeda et al., 2013). The secretory expression of genes encoding α-amylase from *Streptococcus bovis*, β−glucosidase from *Thermobifida fusca*, and β−xylosidase from *B. subtilis* with PorH anchor protein, respectively, in engineered *C. glutamicum* strains enabled efficient utilization of soluble starch, cellobiose, and xylooligosaccharides, respectively, and sustainable production of cadaverine. Methanol is also a sustainable carbon source for microbial fermentations. Introduction of methanol oxidation and formaldehyde assimilation pathways as well as blocking formaldehyde oxidation (Δ*ald*Δ*fadH*) in non-methylotrophic *C. glutamicum* led to cell growth in the presence of methanol and a partial conversion of methanol to cadaverine (Lessmeier et al., 2015). The overall approach for enhanced production of cadaverine from xylose in *C. glutamicum* was performed based on an integrated analysis of fluxome and transcriptome and *in silico* pathway modeling (Buschke et al., 2013). Systems metabolic engineering of *C. glutamicum* targeted to genes related with pentose phosphate pathway, TCA cycle, and by-products formation enabled efficient production of 103 g/L of cadaverine from xylose with a product yield of 32%.

#### 5-Aminovalerate

5-Aminovalerate (5-AVA, 5-aminopentanoic acid) is a non-proteinogenic 5-carbon ω-amino acid and is an attractive building block for polymer synthesis (Rohles et al., 2016; Shin et al., 2016). 5-AVA can be converted to δ-valerolactam *via* a cyclization reaction followed by synthesis of bio-based PA 6,5 from purified δ-valerolactam with ε-caprolactam (Park et al., 2014).

As *C. glutamicum* is a well-established industrial L-lysine producer, the metabolic pathway from L-lysine toward 5-AVA was reconstructed by expressing *davB* and *davA* coding for lysine 2-monooxygenase and 5-aminovalereramidase from *Pseudomonas putida* in several tested L-lysine overproducing strains (Rohles et al., 2016; Shin et al., 2016; Joo et al., 2017). Of these, Joo et al. (2017) achieved 40 g/L of 5-AVA in fed-batch culture from D-glucose. In addition, they examined the feasibility of 5-AVA production (12.5 g/L) from lignocellulosic raw material, a *Miscanthus* hydrolyzate solution. Meanwhile, Rohles et al. (2016) demonstrated that accumulation of the by-products L-lysine and glutarate can be minimized by deleting the *lysE* gene and a putative *gabT* gene, yielding 28 g/L AVA with a productivity of 0.9 g/L/h from L-glucose and beet molasses as carbon sources in fed-batch fermentation. The lysine 2-monooxygenase in the synthetic pathway requires oxygen for activity, which often may limit production performance during fermentation. In this regard, an alternative pathway comprising *ldcC*, *patA*, and *patD* genes encoding lysine decarboxylase, putrescine transaminase, and γ-aminobutyraldehyde dehydrogenase, respectively, from *E. coli* was constructed in *C. glutamicum* combined with blocking the accumulation of the by-products cadaverine, *N*-acetylcadaverine, and glutarate (Jorge et al., 2017b). The resulting strain enabled production of 5.1 g/L 5-AVA with a productivity of 0.12 g/L/h from D-glucose in shake flasks. Also, 5-AVA was produced from the alternative feedstocks starch, glucosamine, xylose, arabinose, and rice straw hydrolyzate (Jorge et al., 2017b; Sasikumar et al., 2021).

### Shikimate-Pathway Derived Nitrogenous Aromatic Compounds

A wide range of aromatic compounds produced from petroleum-based feedstocks and extraction of plants are currently being explored by microbial fermentation (Lee and Wendisch, 2017a). Due to the presence of multiple metabolic pathways that degrade and assimilate various aromatic compounds and its inherently higher tolerance to toxic aromatics, *C. glutamicum* is considered a prominent strain for the production of nitrogenous aromatic compounds, such as halogenated L-tryptophan (Veldmann et al., 2019a,b), *N*-methyl-L-phenylalanine (Kerbs et al., 2021), 4-aminobenzoate, anthranilate, *N*-methylanthranilate (Walter et al., 2020), indole-3-actic acid (Kim et al., 2019), and violacein (Wendisch and Lee, 2020; Figure 2). Recent advances in metabolic engineering of *C. glutamicum* with respect to the sustainable production of nitrogenous aromatic compounds from alternative feedstocks are listed in Table 1.

#### 7-Chloro-L-Tryptophan

Halogenated aromatics are important building blocks or intermediates of bioactive compounds associated with the agrochemical, chemical, and pharmaceutical industries (Payne et al., 2015; Veldmann et al., 2019a,b). Generally, the chemical halogenation processes of arenes often suffer from poor regioselectivity and require harsh reaction conditions. It was known that flavin-dependent halogenase overcomes these issues and catalyzes the halogenation of arene compounds with high regioselectivity (Payne et al., 2015). Of these, 7-chloro-L-tryptophan (7-Cl-Trp) is a precursor of the alkaloid antibiotic rebeccamycin and the antifungal pyrrolnitrin (Veldmann et al., 2019b). Recently, enzymatic halogenation of L-tryptophan was established by utilizing immobilized tryptophan 7-halogenase with cofactor regeneration system (Frese and Sewald, 2015).

Fermentative production of halogenated amino acid 7-Cl-Trp from renewable sources and chloride salts was realized by Veldmann et al. (2019b) in an L-tryptophan-producing *C. glutamicum*. First, to provide a precursor, L-tryptophan-producing *C. glutamicum* was metabolically engineered by introducing genes encoding feedback-resistant enzymes AroG and TrpE and blocking carbon flow to L-tyrosine and L-phenylalanine as well as by plasmid-mediated expression of *E. coli trpD*. Finally, overexpression of the genes encoding tryptophan 7-halogeanse RebH and flavin reductase RebF from *Lechevalieria aerocolonigenes* led to chlorination of L-tryptophan to 7-Cl-Trp (108 mg/L). For access of *C. glutamicum* to renewable feedstocks, *araBAD*, *xylAB*, and *nagB* genes, respectively, were transformed to *C. glutamicum*, resulting in efficient utilization of arabinose, xylose, and glucosamine and production of 7-Cl-Trp in flasks cultivation.

#### *N*-Methyl-L-Phenylalanine

Many bioactive peptides harbor *N*-methylated amino acids, e.g., *N*-methyl-L-alanine, *N*-methyl-L-glutamate, sarcosine, and *N*-methyl-L-phenylalanine (NMePhe). In particular, NMePhe-rich peptides can be considered as promising blood–brain barrier shuttle candidates for improving brain drug delivery of small neurodrugs (Malakoutikhah et al., 2010).

*De novo* production of NMePhe in *C. glutamicum* was based on reductive methylamination of phenylpyruvate by an engineered mutant DpkA^P262A, M141L^, which exhibited similar catalytic efficiencies for phenylpyruvate and pyruvate (Kerbs et al., 2021). To provide phenylpyruvate pool, the carbon flux into L-tryptophan and L-phenylalanine was blocked by removing *trpEG, ilvE*, and *aroT* genes in genome of anthranilate-producing *C. glutamicum*. Upon expression of *dpkA*^P262A, M141L^ and *pheA*^FBR^ encoding feedback-resistant chorismate mutase/prephenate dehydratase from *E. coli*, the engineered stain produced 0.73 g/L of NMePhe from glucose-based fermentation. When genes involved in xylose-utilizing enzymes were expressed in the engineered strain, an NMePhe titer of 0.6 g/L with a productivity of 0.008 g/L/h was reached from xylose-based fermentation.

## Prospects and Conclusion

To better make use of sidestreams from aqua- and agriculture for fermentative processes with engineered *C. glutamicum* strains producing nitrogen-containing value-added compounds we foresee the need for strain optimization and process intensification. ALE has already been applied to improve growth and production with methanol (Hennig et al., 2020; Wang et al., 2020) and has led to a new pathway of fructose utilization (Krahn et al., 2021). Moreover, volumetric productivities for 5-aminovalerate and glutarate have been enhanced by ALE approaches (Haupka et al., 2020; Prell et al., 2021). ALE will facilitate and accelerate the access to various (nitrogen-containing) feedstocks overcoming recalcitrance of the (polymeric) substrate, toxicity of their breakdown products or contaminants they contain.

Hydrolyzates such as spent sulfite liquor (SSL) are challenging to use since they may contain inhibitors and differ between batches. Online off-gas analysis and particle filtering for simultaneous process state and parameter estimation have recently been used to judge performance of a single *C. glutamicum* strain during SSL bioprocessing (Sinner et al., 2021). Furthermore, synthetic consortia have been developed for division of labor between different strains, e.g., various *C. glutamicum* strains or *C. glutamicum* and *E. coli* strains production based on either starch, chitin or SSL (Sgobba et al., 2018b; Pérez-García et al., 2021; Vortmann et al., 2021). SSL bioprocessing for production of riboflavin was improved in fed-batch fermentations based on a inoculation strategy using strains that either utilized glucose, glucose + mannose, glucose + xylose or all three sugars (Pérez-García et al., 2021). Besides using consortia of independent strains of the same species, a mutualistic synthetic *E. coli*/*C. glutamicum* consortium produced lysine, cadaverine and L-pipecolic acid from starch (Sgobba et al., 2018b). The lysine producing, starch-negative *C. glutamicum* strain produced the nitrogenous target compounds from glucose liberated by the lysine auxotrophic, starch hydrolyzing *E. coli* strain that functioned as substrate-converter. Despite the enormous potential that such synthetic microbial consortia have for biotechnological applications, fundamental research questions remain to be answered and process control strategies such as Kalman filtering have to be developed (Tuveri et al., 2021). In this respect, the single-cell behavior has to be studied by dynamic microfluidic single-cell cultivation (dMSCC) technology. As a first application, dMSCC was used for lysine production by a consortium consisting of a lysine auxotrophic strain and a strain producing lysine when induced with IPTG. Notably, the use photocaged IPTG with/without deprotection by light demonstrated at the single-cell level that the lysine auxotrophic strain depended on the lysine producing strain (Burmeister et al., 2021). While insight gained by these approaches still needs to be transferred to real-world fermentative processes, we foresee important contributions of microbial consortia to the field.

Biorefinery processes typically operate under sterile conditions with the associated costs for, e.g., autoclaving. Phosphite (also name phosphonate), which is used as organic fertilizer and is non-toxic to humans and animals, does not support growth of *C. glutamicum* as source of phosphorus. Transfer of phosphite dehydrogenase from *Pseudomonas stutzeri* to a lysine producing *C. glutamicum* strain enabled growth in non-autoclaved growth medium that contained phosphite instead of phosphate (Lei et al., 2021). While lysine was produced under non-sterile conditions, this approach can only be applied at larger scale using sidestreams from aqua- and agriculture that lack phosphate or require addition of a phosphorus source.

## Author Contributions

VW, KN, and J-HL wrote, revised, and edited the manuscript. All authors contributed to the article and approved the submitted version.

## Conflict of Interest

The authors declare that the research was conducted in the absence of any commercial or financial relationships that could be construed as a potential conflict of interest.

## Publisher’s Note

All claims expressed in this article are solely those of the authors and do not necessarily represent those of their affiliated organizations, or those of the publisher, the editors and the reviewers. Any product that may be evaluated in this article, or claim that may be made by its manufacturer, is not guaranteed or endorsed by the publisher.
